# A model for tetrapyrrole synthesis as the primary mechanism for plastid-to-nucleus signaling during chloroplast biogenesis

**DOI:** 10.3389/fpls.2013.00014

**Published:** 2013-02-13

**Authors:** Matthew J. Terry, Alison G. Smith

**Affiliations:** ^1^Centre for Biological Sciences, University of SouthamptonSouthampton, UK; ^2^Institute for Life Sciences, University of SouthamptonSouthampton, UK; ^3^Department of Plant Sciences, University of CambridgeCambridge, UK

**Keywords:** retrograde signaling, photomorphogenesis, heme, protochlorophyllide, chlorophyll, ferrochelatase, *gun* mutants, singlet oxygen

## Abstract

Chloroplast biogenesis involves the co-ordinated expression of the chloroplast and nuclear genomes, requiring information to be sent from the developing chloroplasts to the nucleus. This is achieved through retrograde signaling pathways and can be demonstrated experimentally using the photobleaching herbicide, norflurazon, which in seedlings results in chloroplast damage and the reduced expression of many photosynthesis-related, nuclear genes. Genetic analysis of this pathway points to a major role for tetrapyrrole synthesis in retrograde signaling, as well as a strong interaction with light signaling pathways. Currently, the best model to explain the genetic data is that a specific heme pool generated by flux through ferrochelatase-1 functions as a positive signal to promote the expression of genes required for chloroplast development. We propose that this heme-related signal is the primary positive signal during chloroplast biogenesis, and that treatments and mutations affecting chloroplast transcription, RNA editing, translation, or protein import all impact on the synthesis and/or processing of this signal. A positive signal is consistent with the need to provide information on chloroplast status at all times. We further propose that GUN1 normally serves to restrict the production of the heme signal. In addition to a positive signal re-enforcing chloroplast development under normal conditions, aberrant chloroplast development may produce a negative signal due to accumulation of unbound chlorophyll biosynthesis intermediates, such as Mg-porphyrins. Under these conditions a rapid shut-down of tetrapyrrole synthesis is required. We propose that accumulation of these intermediates results in a rapid light-dependent inhibition of nuclear gene expression that is most likely mediated via singlet oxygen generated by photo-excitation of Mg-porphyrins. Thus, the tetrapyrrole pathway may provide both positive and inhibitory signals to control expression of nuclear genes.

## INTRODUCTION

Chloroplasts are essential organelles in plant cells, responsible for harvesting the majority of the Earth’s energy obtained from the sun. Understanding chloroplast biogenesis is therefore both of great fundamental importance, and is essential in underpinning attempts to manipulate this process in the search for new sources of renewable energy. Chloroplasts evolved through the integration of free-living photosynthetic prokaryotic organisms into eukaryotic hosts, following an endosymbiotic relationship. However, the gene complement of these endosymbionts (encoding as many as 4500 proteins) has since been redistributed so that plant chloroplasts now encode genes for fewer than 100 proteins (Martin et al., 2002), with the remaining genes in the nucleus. As a consequence some 2000–3000 proteins are synthesized in the cytosol, and imported into the chloroplast (Gray et al., 2003; Zybailov et al., 2008; Jung and Chory, 2010). The regulation of chloroplast development and function therefore requires the co-ordination of both nuclear and chloroplast genomes.

**FIGURE 1:**
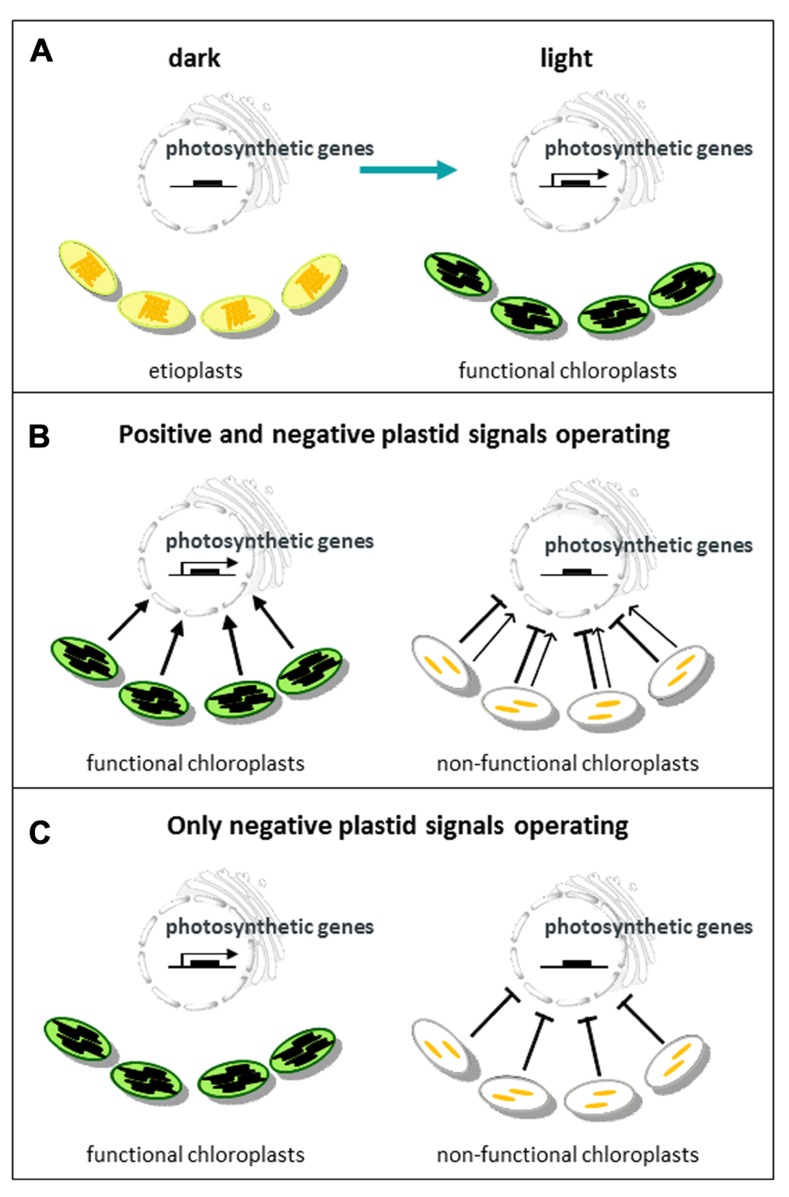
**Models for communication between plastids and the nucleus**. **(A)** During chloroplast biogenesis light promotes the synthesis of many nuclear-encoded proteins required for the development of etioplasts into functional chloroplasts. **(B)** Under normal conditions a positive plastid signal provides information on the developmental status of the chloroplast and promotes nuclear gene expression. Chloroplast damage or loss of function leads to a reduction in the positive signal (in most conditions) and an additional inhibitory signal. **(C)** The alternative model in which only an inhibitory plastid signal resulting from chloroplast damage or loss of function provides information, but there is no feedback from the chloroplast to the nucleus under normal conditions.

There are two major groups of chloroplast-targeted proteins encoded by the nucleus: important components of the chloroplast genetic machinery, including one of the RNA polymerases and a large number of pentatricopeptide repeat (PPR) proteins involved in RNA processing; and the enzymes and other nucleus-encoded chloroplast proteins that comprise the components of the photosynthetic machinery. This latter group, referred to as “photosynthetic genes” (**Figure 1A**) are expressed in response to light via anterograde signaling pathways, which include those mediated by the phytochrome and cryptochrome families of photoreceptors (Waters and Langdale, 2009). Since many components of these signaling pathways are shared with other de-etiolation responses, considerable progress has been made recently in understanding light regulation of anterograde signaling (Leivar et al., 2009; Shin et al., 2009; Stephenson et al., 2009; Richter et al., 2010).

However, in any regulatory system information needs to travel in both directions, and chloroplasts are able to send information back to the nucleus to control expression of photosynthetic genes via plastid-to-nucleus signaling (hereafter termed plastid signaling). In mature plants, chloroplasts can provide the nucleus with information about the environment that serves to optimize photosynthesis and other aspects of plant function such as the response to a variety of stresses. This type of plastid signaling has been defined as *operational control* (Pogson et al., 2008). This article, however, is concerned with *biogenic control*, i.e., signals that provide information about the developmental status of the chloroplast during the process of chloroplast biogenesis. Just as importantly these signals will also provide information on the number of developing chloroplasts that need to be provided with new proteins. Biogenic control has been studied primarily in de-etiolating seedlings, but may equally apply during the synthesis of new chloroplasts in the apical meristem. However, in addition to these developmental signals a young seedling is exposed to the same environmental cues as mature plants and extremes of light and temperature are likely to have even more severe effects at this stage of development. Therefore models of plastid signaling during biogenic control need to include integration of environmental information. So what would be the nature of such a signal? If a signal is required to provide information on the progression of chloroplast development then it makes sense that this signal is present throughout the developmental process. Only a positive plastid signal, as illustrated in **Figure 1B**, can provide this continuous information. An inhibitory signal that provides information about chloroplast damage should not be continuous – the only information potentially provided by a negative signal produced under normal conditions is its absence (**Figure 1C**). There is evidence in the early literature for a continuous plastid signal that permits expression of light-regulated genes (Oelmüller et al., 1986; Burgess and Taylor, 1988), and as we shall see such a model is now supported genetically (Woodson et al., 2011).

It is over 30 years since Bradbeer et al. (1979) first inferred that plants with abnormal chloroplasts showed reduced nuclear gene expression and thus that signals from the chloroplast were required for the expression of certain nuclear genes. However, despite remaining a highly active research area, our understanding of how chloroplasts signal to the nucleus has remained opaque (Pogson et al., 2008; Jung and Chory, 2010; Kleine et al., 2009; Pfannschmidt, 2010; Inaba et al., 2011; Leister, 2012). There are a number of reasons for this. Research on plastid signaling has been limited by the fact we have only isolated data sets utilizing different experimental systems. The use of different species, plastid treatments, seedling age, gene outputs etc., has contributed to confusion in the field that has hampered the development of robust and testable models for plastid signaling. A further complication is that the severity of treatments used invariably elicits a strong stress response that may mask signaling responses under normal physiological conditions, including the adjustment to mild stress conditions. These problems are particularly pertinent when trying to separate the role of plastid signaling during chloroplast biogenesis from that of mature chloroplasts. In this article we propose a model for plastid signaling during chloroplast biogenesis in which there are positive and negative plastid signals regulating nuclear gene expression. In doing this we have synthesized the available data to examine the role of tetrapyrrole synthesis in mediating both of these plastid signals.

## JUST HOW MANY PLASTID SIGNALING MOLECULES ARE THERE?

Retrograde signaling was demonstrated in early experiments using plants with defective plastids, either as a result of mutation (Harpster et al., 1984) or the use of inhibitors of plastid translation or norflurazon (NF), a photobleaching herbicide that inhibits the carotenoid synthesis enzyme phytoene desaturase (Oelmüller et al., 1986). These experiments showed that plastid integrity was required for the expression of nuclear-encoded photosynthetic genes. Indeed, NF and the plastid translation inhibitor lincomycin (Lin) are still the standard experimental tools today for demonstrating the requirement for functional chloroplasts to maintain nuclear gene expression during chloroplast biogenesis. NF treatment results in photo-oxidative damage of chloroplasts under white light due to unquenched triplet chlorophyll formation and leads to a catastrophic reduction in the expression of nearly 1000 nuclear genes (Strand et al., 2003; Koussevitzky et al., 2007; Moulin et al., 2008; Aluru et al., 2009). Lin treatment results in an even stronger response, with twice as many genes down-regulated (Cottage et al., 2008; Ruckle et al., 2012), provided that it is applied within 2–3 days of germination (Oelmüller et al., 1986; Gray et al., 2003).

Since the earliest observations the search has been on for the chloroplast-derived molecules that are affected by these treatments and thus could be signaling the nucleus to bring about changes in nuclear gene expression. There are now a range of candidate molecules in the literature (most recently reviewed by Leister, 2012), many of them coming to light quite recently. They can be categorized broadly into three separate classes of molecule:

### PROTEINS

There are two proteins that have been shown recently to be translocated between plastids and the nucleus, suggesting they may be good candidates for mediating signaling between these compartments. Introduction of the gene for the transcriptional activator Whirly1 into tobacco chloroplast DNA resulted in synthesis of the protein in the chloroplast, but it was able to translocate to the nucleus to activate pathogen response genes (Isemer et al., 2012). In the second, the chloroplast envelope-bound plant homeodomain transcription factor (PTM) was shown to undergo proteolytic cleavage under conditions affecting plastid signaling, resulting in the accumulation of the amino terminal fragment in the nucleus (Sun et al., 2011). This PTM fragment targets the *ABI4* gene, activating its expression and, since ABI4 itself is implicated in plastid-dependent regulation of nuclear gene expression (Koussevitzky et al., 2007), this signaling mechanism looks very promising.

### REACTIVE OXYGEN SPECIES

Reactive oxygen species (ROS) can be generated in the chloroplast through the action of photosynthesis with the superoxide anion radical and hydrogen peroxide produced from the reduction of oxygen at photosystem I (PSI) and singlet oxygen produced at PSII (Apel and Hirt, 2004). Accumulation of all three species has been shown to result in major changes in nuclear gene expression and thus convey information about the status of the chloroplast (op den Camp et al., 2003; Gadjev et al., 2006; Galvez-Valdivieso and Mullineaux, 2010).

### METABOLITES

Several different classes of metabolites have the potential to be involved in chloroplast-to-nucleus communication. They are characterized by being synthesized in the chloroplast and then translocated into the cytosol or to other cellular compartments. Molecules such as amino acids, lipids, and reducing equivalents are exported from the chloroplast, and many hormones including the gibberellins, abscisic acid (ABA), jasmonate, and the strigolactones are at least partially synthesized in the chloroplast. One group of molecules that has been repeatedly implicated is the tetrapyrroles (discussed in more detail below). Heme, the phytochrome chromophore phytochromobilin, and chlorophyll breakdown products, are all known to leave the chloroplast and thus have the potential to modify cellular processes (Mochizuki et al., 2010). Other metabolites have also been shown recently to affect nuclear gene expression. The isoprenoid precursor methylerythritol cyclodiphosphate (MEcPP) was suggested as a plastid-derived signal regulating the nuclear gene *HPL* encoding the chloroplast-localized oxylipin biosynthesis enzyme hydroperoxide lyase (Xiao et al., 2012). Another example, is 3^′^-phosphoadenosine 5^′^-phosphate (PAP), which has been demonstrated to play a role as a chloroplast signal regulating a number of drought and high light-inducible genes, including *APX2* (Estavillo et al., 2011).

Can any of these signals result in the dramatic reduction in nuclear gene expression seen after severe treatments, such as with NF or Lin? One hypothesis is that there is not a single signal, and with so many chloroplast-derived metabolites able to influence nuclear gene expression, it is the combination of their effects – as metabolite signatures – that we observe as a plastid signal (Pfannschmidt, 2010). While this is a useful concept in our understanding of signal integration, it cannot account for the precipitous reduction in expression of many nuclear genes when chloroplast development is blocked. Such a proposal is also inconsistent with transcriptomic meta-analyses (Richly et al., 2003; Biehl et al., 2005). In these studies an analysis of the regulation of an almost complete gene set of nucleus-encoded chloroplast genes indicated that there was one master regulatory switch leading to the up- or down-regulation of a conserved group of genes and that this was tightly linked to mutations affecting the chloroplast (Richly et al., 2003). A more exhaustive follow-up study supported this idea, but was able to sub-divide gene classes further by function (Biehl et al., 2005), suggesting differential interaction with other signals. Regulation through the combined signaling of many metabolites might be expected to show a more complex gene expression profile. Since these studies were conducted with mature plants it suggests that a limited number of specific signals are also likely to be important during operational plastid signaling. However, signals such as MEcPP or PAP may reflect the response to changes in environmental conditions not tested for in these meta-analyses and could still contribute to a broad spectrum of signals involved in operational control, interacting with other signals such as those related to redox state (Dietz and Pfannschmidt, 2011). To drive the formation and development of chloroplasts during biogenesis it is more likely that a limited number of specific signals are required. And to understand this biogenic control we need to consider fully the genetic evidence available.

## THE GENETICS OF PLASTID SIGNALING DURING CHLOROPLAST BIOGENESIS (*GENOMES UNCOUPLED* MUTANTS)

There are very many mutations and treatments affecting chloroplast function that lead to a reduction in the expression of nuclear genes (discussed in more detail below). However, what we currently know about the pathways regulating chloroplast-to-nucleus communication during chloroplast biogenesis comes primarily from the isolation of *genomes uncoupled *(*gun*) mutants in which the expression of the nuclear gene *Lhcb* is maintained following chloroplast damage using NF treatment (Susek et al., 1993). To date, screening for this phenotype has generated mutants in two major categories: mutants affected in tetrapyrrole metabolism (Susek et al., 1993; Mochizuki et al., 2001; Larkin et al., 2003; Woodson et al., 2011) and mutants in light signaling components (Ruckle et al., 2007). Given the close association between light and plastid-signaling (Vinti et al., 2005; Larkin and Ruckle, 2008), and the primary role of light in regulating chloroplast development (Waters and Langdale, 2009), it is perhaps not surprising that a mutant lacking the blue-light photoreceptor cryptochrome 1 (CRY1) and the light signaling mutants *hy5* and *cop1* show altered response to chloroplast status (Ruckle et al., 2007). HY5 is a basic leucine zipper transcription factor that normally induces the expression of photosynthesis-related genes in response to phytochrome and cryptochrome signaling. However, it has been proposed that HY5 is converted to a negative regulator of photosynthetic genes after Lin treatment (Ruckle et al., 2007) when its function is dependent on the presence of another transcription factor ABI4 (Larkin and Ruckle, 2008). Consistent with this, *abi4* mutants also show a *gun* phenotype (Koussevitzky et al., 2007). Integration of light and plastid signaling responses at the genetic level supports earlier observations that the *cis*-regulatory elements mediating these responses appear to be common to both processes (Bolle et al., 1994; Kusnetsov et al., 1996; Puente et al., 1996; McCormac et al., 2001).

**FIGURE 2:**
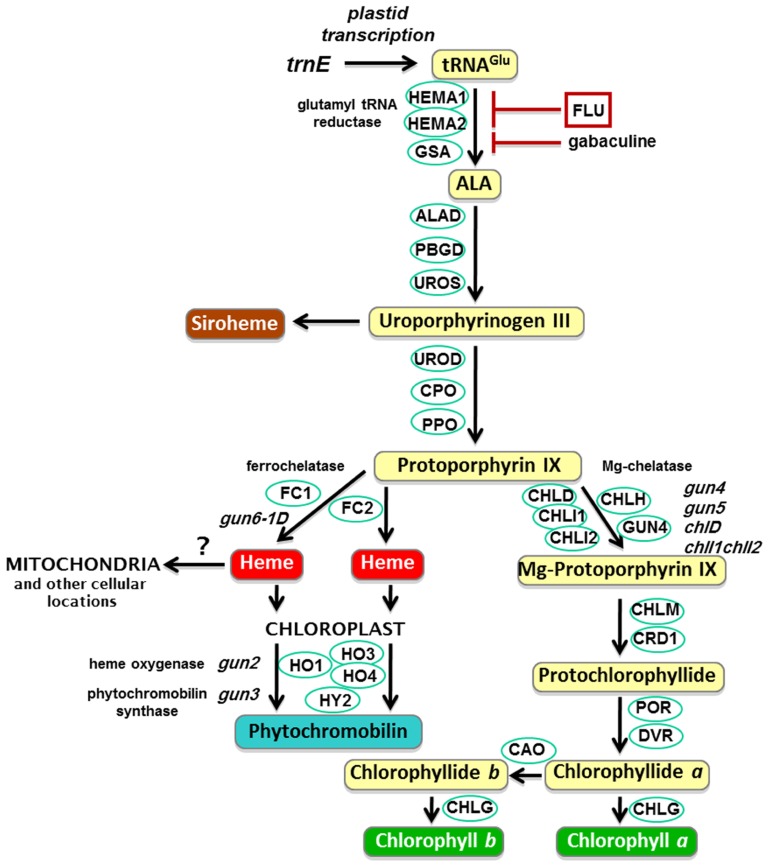
**The plant tetrapyrrole pathway with key enzymes and *gun* mutants**. ALA, 5-aminolevulinic acid; HEMA, glutamyl-tRNA reductase; GSA, glutamate-1-semialdehyde 2,1-aminomutase; ALAD, 5-aminolevulinate dehydratase; PBGD, porphobilinogen deaminase; UROS, uroporphyrinogen III synthase; UROD, uroporphyrinogen III decarboxylase; CPO, coproporphyrinogen III oxidase; PPO, protoporphyrinogen IX oxidase; CHLH, Mg-chelatase H subunit; CHLI, Mg-chelatase I subunit; CHLD, Mg-chelatase D subunit; GUN4, regulator of Mg-chelatase; CHLM, Mg-proto IX methyltransferase; CRD1, Mg-proto IX monomethylester cyclase; POR, NADPH:protochlorophyllide oxidoreductase A, B and C; DVR, divinyl-protochlorophyllide reductase; CHLG, chlorophyll synthase; CAO, chlorophyllide *a* oxygenase; FC, ferrochelatase; HO, heme oxygenase; HY2, phytochromobilin synthase.

The original *gun* mutant screen isolated five mutants that retained partial expression of *Lhcb *after NF treatment. GUN1 is a PPR protein that binds nucleic acids (Susek et al., 1993; Koussevitzky et al., 2007) and is discussed later. The other four *gun* mutants were found to be mutated in tetrapyrrole synthesis genes: *GUN2*, *GUN3*, and *GUN5* encode heme oxygenase, phytochromobilin synthase, and the H subunit of Mg-chelatase (CHLH), respectively (**Figure 2**; Mochizuki et al., 2001), while GUN4 is a regulator of Mg-chelatase activity (Larkin et al., 2003). Subsequently it has been demonstrated that mutants lacking the D subunit (Strand et al., 2003) and both I subunits (Huang and Li, 2009) of Mg-chelatase also show a *gun* phenotype. Based on the characterization of these mutants and the apparent observation that the chlorophyll biosynthetic intermediate, Mg-protoporphyrin IX (Mg-proto), accumulated after NF treatment, it was proposed that Mg-proto acted as a mobile signal mediating chloroplast regulation of nuclear gene expression (Strand et al., 2003). However, this result has not been supported by further biochemical and genetic analysis in seedlings (Gadjieva et al., 2005; Mochizuki et al., 2008; Moulin et al., 2008; Voigt et al., 2010). In these experiments detailed measurements of Mg-proto and its methyl ester showed that there was no correlation between levels of these chlorophyll precursors and nuclear gene expression when Mg-proto levels were manipulated genetically (Mochizuki et al., 2008; Voigt et al., 2010) or using a range of growth conditions and treatments (Moulin et al., 2008). Moreover, a liquid chromatography-mass spectrometry approach was used to identify unambiguously the intermediates being measured, and no large accumulation of Mg-proto was observed under any condition, further supporting this conclusion (Moulin et al., 2008). These results also explained a previous observation that the *xantha-l* mutant of barley that accumulates Mg-proto did not show a reduction in nuclear gene expression (Gadjieva et al., 2005). Instead the identification of a dominant *gun* mutation (*gun6-1D*) that results in the overexpression of ferrochelatase-1 (FC1) has led to a model in which flux through a specific heme pool mediates plastid signaling (Woodson et al., 2011). This model provides an explanation for the phenotype of all the tetrapyrrole-related *gun* mutants in the tetrapyrrole pathway. As can be seen from **Figure 2**, blocking Mg-chelatase activity would be expected to direct protoporphyrin IX to heme synthesis (Cornah et al., 2003) thus increasing FC1 activity and heme levels. Similarly, inhibition of heme degradation in the *gun2* and *gun3* mutants would also be predicted to increase heme. Importantly, the simplest interpretation of the FC1-dependent *gun* phenotype is that it corresponds to the production of a positive signal promoting expression of nuclear-encoded chloroplast genes (**Figure 1B**) – the first such direct genetic evidence.

## A HEME-MEDIATED POSITIVE PLASTID SIGNAL DURING CHLOROPLAST BIOGENESIS

The observation that the dominant *gun6 *mutant had a *gun* phenotype was confirmed by overexpression of FC1 in wild-type plants. In contrast, overexpression of a second ferrochelatase isoform (FC2; **Figure 2**) did not result in this phenotype (Woodson et al., 2011). Moreover, a catalytically inactive FC1 was also unable to rescue nuclear gene expression, and the role of FC1 was dependent on flux through the pathway. These results indicate that it is the activity of FC1 that is crucial, and suggest that a specific FC1-derived pool of heme is functioning as a positive signal (or the precursor of such a signal) to promote expression of nuclear-encoded photosynthetic genes (Woodson et al., 2011). Furthermore, overexpression of FC1 can also rescue the loss of nuclear gene expression observed in a mutant lacking the SIG2 sigma factor involved in plastid-encoded RNA polymerase (PEP)-dependent plastid transcription (Woodson et al., 2013).

Does the model of a positive heme-related signal fit with all of the current data? Strand et al. (2003) originally reported that mutants in two trunk pathway enzymes, porphobilinogen deaminase (PBGD) and *lin2* lacking coproporphyrinogen oxidase (CPO; see **Figure 2**), showed a *gun* phenotype, as did treatment with the protoporphyrinogen oxidase (PPO) inhibitor S23142, results that would be inconsistent with heme as a positive regulator. In contrast, Woodson et al. (2011) reported that *lin2* did not show a *gun* phenotype, and that neither did the *hema1hema2* double mutant, defective in glutamyl-tRNA reductase, a key enzyme in the synthesis of the tetrapyrrole precursor 5-aminolevulinic acid (ALA), nor treatment with gabaculine, which inhibits the second ALA synthesis enzyme, glutamate semialdehyde aminomutase. Voigt et al. (2010) also failed to detect a *gun* phenotype for a range of mutants in enzymes in the trunk tetrapyrrole pathway between ALA and protoporphyrin IX. Clearly a consensus needs to be reached on the characterization of these mutants. Similarly, it is difficult to reconcile the observation that ALA feeding can inhibit gene expression in dark (D) and far-red light (FR) grown seedlings (Vinti et al., 2000). A more recent study confirmed this result, but showed that ALA can rescue nuclear gene expression when the heme signal appears to be limiting, such as in the *sig2* mutant (Woodson et al., 2013). The demonstration that overexpression of protochlorophyllide oxidoreductase A and B (PORA and PORB) also causes a *gun* phenotype (McCormac and Terry, 2002) can most probably be explained by the extraordinary photo-oxidative buffering capacity of these proteins, which could have reduced the impact of the NF treatment. Alternatively, as POR overexpression also inhibits a proposed inhibitory pathway in the light (see later), this may have contributed to the observed phenotype on NF.

Another area that requires further resolution is the measurement of the proposed heme pool. Heme is present in plant cells bound covalently to c-type cytochromes, and non-covalently to b-type cytochromes and the vast majority of other hemoproteins, which include cytochromes P450, nitrate reductase, NADPH oxidases, peroxidases, and catalases (Cornah et al., 2003; Mochizuki et al., 2010). It is generally assumed that there is a pool of “free” heme that is either in transit between compartments, or associated with the site of synthesis, although since heme is very lipophilic it is unlikely to be in solution. It is this free-heme pool that is proposed to be involved in signaling, and it is likely to be small compared with total cellular heme. However, there is currently no satisfactory way of measuring free heme, nor even confirming that it is present. Woodson et al. (2011) measured total non-covalently bound heme by acid-acetone extraction and a chemiluminescence-based detection method using reconstitution of horseradish peroxidase activity (Masuda and Takahashi, 2006), and found it was reduced after NF treatment, but not measurably increased in the *gun* mutants (with the exception of the heme oxygenase-deficient *gun2*). It is worth noting though, that *gun1* and *gun5* could both rescue heme levels in the *sig2* mutant background, which also had reduced heme (Woodson et al., 2013). The reduction in total non-covalently bound heme after a NF treatment (Woodson et al., 2011) is in agreement with an earlier acid extraction-based measurement (Kumar et al., 1999), but measurement of heme using alkali-acetone extraction and the same chemiluminescence-based detection assay produced the opposite result (Voigt et al., 2010). A more thorough analysis of this problem using a combination of different extraction methodologies suggests that a neutral-acetone extraction may give the best approximation of an unbound heme pool (Espinas et al., 2012). In this study, although total heme was reduced after NF treatment, an increase in an unbound free heme pool was observed, indicating that the method used by Voigt et al. (2010) more closely approximated to the measurement of an unbound heme pool. Nonetheless, how this information relates to a putative heme signal remains unclear. Any signal could be transient and may not accumulate at all, being instantly metabolized to a bilin for example. Certainly, there is no requirement for heme to be a mobile signal. Indeed, although heme is significantly less photo-toxic than Mg-proto, it is still a reactive molecule that interacts with other cellular components and thus any heme functioning as a signaling molecule is likely to be carefully chaperoned.

One potentially important aspect of the data of Woodson et al. (2011) is that an FC1-generated heme pool is associated with a requirement for non-photosynthetic heme. Both *FC1* and *HEMA2* show regulatory patterns inconsistent with a major role in photosynthesis (Ujwal et al., 2002; Nagai et al., 2007), and in fact are induced under stress conditions (Nagai et al., 2007; Moulin et al., 2008; Avin-Wittenberg et al., 2012). Following NF treatment neither gene was repressed, in stark contrast to all other tetrapyrrole synthesis genes except the mitochondrial *PPO2* (Moulin et al., 2008). Interestingly, nuclear gene expression on NF was rescued not only by overexpression of FCI, but also by HEMA2 (Woodson et al., 2011). It has long been speculated that there may be two spatially separated ALA pools providing tetrapyrroles for different purposes (Huang and Castelfranco, 1990), and recent data describing the discovery of a glutamyl-tRNA reductase binding protein (GBP) has put this suggestion back on the agenda (Czarnecki et al., 2011). In this case mutants lacking GBP showed differential inhibition of heme and chlorophyll synthesis, suggesting the possibility of a bifurcated pathway originating from ALA, although the model does not propose a simple separation of *HEMA1*- and *HEMA2*-encoded glutamyl-tRNA reductases. A more sophisticated model of tetrapyrrole synthesis may be needed to account for all the observations reported.

The close association between chloroplast and mitochondrial function has led to the integration of mitochondria into models for chloroplast-to-nucleus communication (Leister, 2005; Pfannschmidt, 2010). For example, in adult plants inhibition of chloroplast and mitochondrial translation had a synergistic effect on the down-regulation of nuclear-encoded chloroplast genes (Pesaresi et al., 2006). In the context of the current discussion it is interesting that mitochondria are a major sink for non-photosynthetic heme for the respiratory complexes. Heme is either made in the chloroplast and then transported to mitochondria, or possibly an earlier precursor is translocated, with the final two steps in heme synthesis taking place in the mitochondria (discussed in Mochizuki et al., 2010). It is tempting to speculate that an FC1-dependent positive plastid signal may require, at least in part, processing in mitochondria. Such a result might account for the synergistic effect of damaging both compartments (Pesaresi et al., 2006), while explaining why chloroplast damage can lead to a loss of gene expression when mitochondrial damage alone does not. The observation that mitochondrial-dependent up-regulation of *ALTERNATIVE OXIDASE1a* is mediated by ABI4 (Giraud et al., 2009), which is strongly implicated in plastid signaling (Koussevitzky et al., 2007), is also intriguing.

## HEME AS A SIGNALING MOLECULE IN OTHER ORGANISMS

So how suitable is heme as a signaling molecule? In fact heme has been implicated in signaling in a wide range of systems and is well established as a signaling molecule in heterotrophic bacteria, fungi, and animals. In these systems, heme is either the only, or the major, tetrapyrrole molecule that is synthesized, and its role in regulating its own synthesis has been intensively investigated. Furthermore, it has also been shown that heme regulates cellular processes more generally, although the exact nature of its role as a regulator varies between organisms (Mense and Zhang, 2006; Furuyama et al., 2007). Nevertheless, evidence has emerged for conserved mechanisms of heme regulation that may be important in our understanding of heme signaling in plants.

In rhizobia and other α-proteobacteria, the iron response regulator (IRR) modulates the cellular response to available levels of iron including regulation of heme synthesis (Small et al., 2009). Under iron-deficiency IRR inhibits expression of the *hemB* gene encoding ALA dehydratase (ALAD; **Figure 2**), thus preventing accumulation of phototoxic heme biosynthesis intermediates. In *Bradyrhizobium japonicum* this is regulated by the conditional stability of IRR, which accumulates under iron-deficient conditions. In iron-replete conditions IRR is bound by ferrochelatase and is directly targeted for degradation by the heme product of this enzyme (Qi and O’Brian, 2002). Therefore in this system heme can act as a signaling molecule without accumulation as free heme. Under iron deficiency protoporphyrin IX accumulates, which promotes dissociation of ferrochelatase from IRR, thus preventing degradation. Deletion studies on IRR from *Bradyrhizobium* showed that it contained two heme binding sites: a heme-regulatory motif (HRM), which is characterized as containing a conserved Cys-Pro, and a second site with a His-xxx-His configuration that bind to ferric and ferrous heme, respectively (Yang et al., 2005a). Interestingly, other rhizobia only contain the His domain and in *Rhizobium leguminosarum* binding of heme to IRR does not affect stability, but instead prevents binding to regulatory promoter sequences (Singleton et al., 2010).

The HRM motif is critical for heme signaling in a wide variety of eukaryotic systems. The first recognition of the HRM came from the study of the mammalian enzyme 5-aminolevulinic acid synthase (ALAS), which in animals and yeast is responsible for the synthesis of ALA in a single step from succinyl coA and glycine (in contrast to the pathway from glutamate involving glutamyl-tRNA reductase and glutamate semialdehyde aminomutase found in plants, algae, and the majority of bacteria). In mammals there are two ALAS isoforms, both of which are targeted to the mitochondria via N-terminal targeting peptides that contain two copies of the HRM consensus sequence (Lathrop and Timko, 1993). Binding of heme to these two HRMs inhibits import of the ALAS precursors into mitochondria *in vitro* although additional factors were required to inhibit import of ALAS2 *in vivo* (Munakata et al., 2004). Another important example relevant to the current discussion comes from the yeast, *Saccharomyces cerevisiae. *In this case, the oxygen-dependent synthesis of heme, via the enzymes CPO and PPO (see **Figure 2**), initiates mitochondrial biogenesis as a result of heme-regulated expression of many nuclear genes (Zhang and Hach, 1999). This is achieved via heme activator protein 1 (HAP1), which contains seven HRMs, with heme binding affecting both the DNA binding and transcription-activation activities of HAP1 (Zhang and Guarente, 1995). Heme also affects nuclear gene transcription in mammals. An important mediator of this process is the basic leucine zipper protein Bach1, which contains six HRM motifs (Ogawa et al., 2001). Bach1 forms heterodimers with the Maf-related oncogene family to repress genes such as *HO-1* encoding heme oxygenase 1. In the presence of heme, Bach1 is ubiquitinated and degraded, leading to increased* HO-1* expression (Zenke-Kawasaki et al., 2007). As well as transcriptional effects, heme also modulates gene expression at the translational level. In rat reticulocytes, under heme-deficient conditions, a heme-regulated inhibitor, eIF2α kinase (HRI) phosphorylates eIF2α, preventing it from being recycled and thus protein synthesis is inhibited (Bauer et al., 2001). When heme is present, it can bind to HRI (which contains two HRMs) inactivating the kinase activity.

A more general role for heme as a signaling molecule has been established from the study of the circadian clock and how it interacts with metabolism. Heme biosynthesis is circadian-regulated and several components of the mammalian clock bind heme including PER2, NPAS2 (Kaasik and Lee, 2004; Yang et al., 2008), and Rev-erbα (Yin et al., 2007). This in turn regulates the ability of these factors to interact with nuclear transcription factors, thus influencing gene expression. In the case of PER2, heme-binding is via an HRM, but for NPAS2 and Rev-erbα, although the axial ligand is still cysteine, it is not within a classic HRM (Shimizu, 2012). Rev-erbα additionally utilizes a histidine as an axial ligand (Yin et al., 2007) and for NPAS2 the cysteine resides in a PAS domain (named after the proteins Per, ARNT, and Sim), a widely occurring domain that functions in binding a wide variety of small molecules (Henry and Crosson, 2011). Another broad role for heme regulation comes from the observation that a key miRNA processing enzyme in human cells, the RNA-binding protein DiGeorge critical region 8 (DGCR8) requires bound heme for activity (Faller et al., 2007). Since up to 30% of all human genes are regulated by miRNAs (Lewis et al., 2005), this indicates the scope for the influence of heme in these cells.

Are these mechanisms directly applicable to photosynthetic systems, where chlorophyll is the major tetrapyrrole synthesized? In addition to the evidence for a role for heme in plastid signaling in *Arabidopsis* (Woodson et al., 2011), heme has also been proposed (along with Mg-proto) as a signal in the green alga *Chlamydomonas reinhardtii* (von Gromoff et al., 2008). Analysis of the *Chlamydomonas* transcriptome showed that the expression of hundreds of genes was affected by heme, but only a few of these genes were associated with a photosynthetic function (Voss et al., 2011). The mechanism for this regulation is unknown and, although heme-binding proteins have been identified in plants (Takahashi et al., 2008; Mochizuki et al., 2010), classic HRM-containing regulators have not. In this context the recent identification of a heme regulatory protein in purple bacteria may be important (Yin et al., 2012). PpsR, which together with AppA mediates light and redox regulation of bacteriochlorophyll biosynthesis in *Rhodobacter sphaeroides*, shows modified DNA binding in response to heme, with heme binding mediated by an atypical HRM with a Cys-Ile motif (Yin et al., 2012). As PAS domains, which also function as heme-binding domains (Gilles-Gonzalez and Gonzalez, 2005), are widespread in plants, and with new regulatory heme-binding motifs being discovered, there is still plenty of potential for direct heme-regulatory mechanisms in plants.

## THE HEME-MEDIATED SIGNAL IS THE PRIMARY PLASTID SIGNAL DURING CHLOROPLAST BIOGENESIS

If we accept that a heme-related signal is the leading candidate as a positive plastid signal based on the genetic evidence, then is this the only signal during chloroplast biogenesis? From the earliest studies it was established that inhibition of chloroplast translation resulted in an inhibition of nuclear gene expression (Oelmüller et al., 1986; Sullivan and Gray, 1999) and the interaction of the translational inhibitor Lin and NF with the *gun* mutants has led to the suggestion that whilst the two treatments both result in disrupted chloroplasts, they affect separate signaling pathways. This was most obviously demonstrated by the observation that inhibition of *Lhcb* expression after Lin treatment is rescued in the *gun1* mutant, but not in the *gun2–gun5* mutants (Gray et al., 2003; McCormac and Terry, 2004; Koussevitzky et al., 2007). Indeed, initial genetic (Mochizuki et al., 2001) and gene expression studies (Strand et al., 2003; McCormac and Terry, 2004) also suggested separate signaling pathways. For example, it was shown that loss of GUN5 had a stronger effect on expression of *Lhcb* than on *HEMA1*, but that this sensitivity was reversed in *gun1* (McCormac and Terry, 2004). However, recent experiments demonstrating that 89% of genes that are repressed by *gun1* are also repressed by *gun5,* and that strong alleles of *gun1* are epistatic to *gun5* (Koussevitzky et al., 2007), support the proposal that they do in fact reside in the same pathway, and that GUN1 plays a central role in all plastid signals including those defined by GUN2–5 (Koussevitzky et al., 2007). If this is the case, then the observation that *HEMA1* expression is fully rescued in a *gun1gun5* double mutant (McCormac and Terry, 2004) supports the idea that the heme signal is the primary, if not the only, positive plastid signal during chloroplast biogenesis.

There are actually many different treatments and mutations that affect chloroplast function and development and have an impact on nuclear gene expression (see Inaba et al., 2011 for a comprehensive list), and the consequences of these impairments have often been interpreted as defining independent signaling pathways. The expression of nucleus-encoded photosynthesis genes is blocked by conditions that inhibit early chloroplast development such as chloroplast transcription inhibitors (Rapp and Mullet, 1991) or mutations affecting transcription (*sig2*; Woodson et al., 2013), plastid RNA editing (Kakizaki et al., 2012), chloroplast protein synthesis (Hess et al., 1994; Pesaresi et al., 2006) or import of nucleus-encoded chloroplast proteins (*ppi2*; Kakizaki et al., 2009). In addition, various mutants with impaired chloroplast development such as the *cue* mutants of *Arabidopsis* also fall into this category (López-Juez et al., 1998; Vinti et al., 2005). It seems unnecessarily complex and therefore rather unlikely that inhibition of these processes each leads to an independent plastid signal, and the observation that *gun1* can rescue the plastid signaling response in *ppi2* (Kakizaki et al., 2009) and *sig2* (Woodson et al., 2013) as well as after Lin and NF treatments, supports the concept of a single primary pathway. We therefore propose that the simplest explanation for the effects of all of the treatments and mutations described above is that they compromise the production and/or processing of the positive heme-related signal.

## A Mg-PORPHYRIN-DEPENDENT INHIBITORY PATHWAY DURING DEFECTIVE CHLOROPLAST DEVELOPMENT

Does a regulatory system in which there is a single positive signal provide the flexibility of regulation required for modulating such a complex and important process as chloroplast biogenesis? Perhaps more specifically if the signal is based on the synthesis of a tetrapyrrole, what happens when tetrapyrrole synthesis is in excess? Overaccumulation of tetrapyrroles has damaging photo-oxidative consequences for a seedling. This is most dramatically demonstrated in the *flu* mutant of *Arabidopsis* (Meskauskiene et al., 2001). FLU is a repressor of glutamyl-tRNA reductase activity and *flu* mutants therefore accumulate high levels of protochlorophyllide in the dark. On transfer to white light there is rapid production of singlet oxygen (op den Camp et al., 2003) resulting in severe tissue damage and seedling death (Wagner et al., 2004). A similar situation is observed in the block-of-greening response induced by a FR treatment prior to transfer to white light (Barnes et al., 1996). During the FR treatment phyA-dependent photoreceptor signaling pathways are activated, but photoconversion of protochlorophyllide by POR (**Figure 2**) does not proceed, resulting in the accumulation of protochlorophyllide at the same time as there is depletion of POR (Barnes et al., 1996; McCormac and Terry, 2002). Such an FR treatment can be lethal, but overexpression of PORA or PORB is able to rescue this response (Sperling et al., 1997). The advantage of this system is that the severity of the treatment can easily be adjusted by the length or fluence rate of the FR period and we have demonstrated that under conditions in which seedlings survive there is severe reduction in nuclear gene expression for photosynthesis-related genes (McCormac and Terry, 2002). Thus, under conditions in which ALA synthesis, and therefore all tetrapyrrole synthesis including a positive, heme-related signal is increased, there is inhibition of nuclear gene expression. This is strongly suggestive of an additional repressive signal. The generation of this signal is inhibited by overexpression of POR in the plastids as well as by the *gun5* mutant in which Mg-proto synthesis is impaired (McCormac and Terry, 2002, 2004). Consistent with it being a separate signal, down-regulation after a FR pre-treatment can be shown to be additive with inhibition of gene expression on NF (McCormac and Terry, 2002). Interestingly though, the FR-induced signal is exacerbated in a *gun1* mutant and inhibited by a simultaneous Lin treatment (McCormac and Terry, 2004).

**FIGURE 3:**
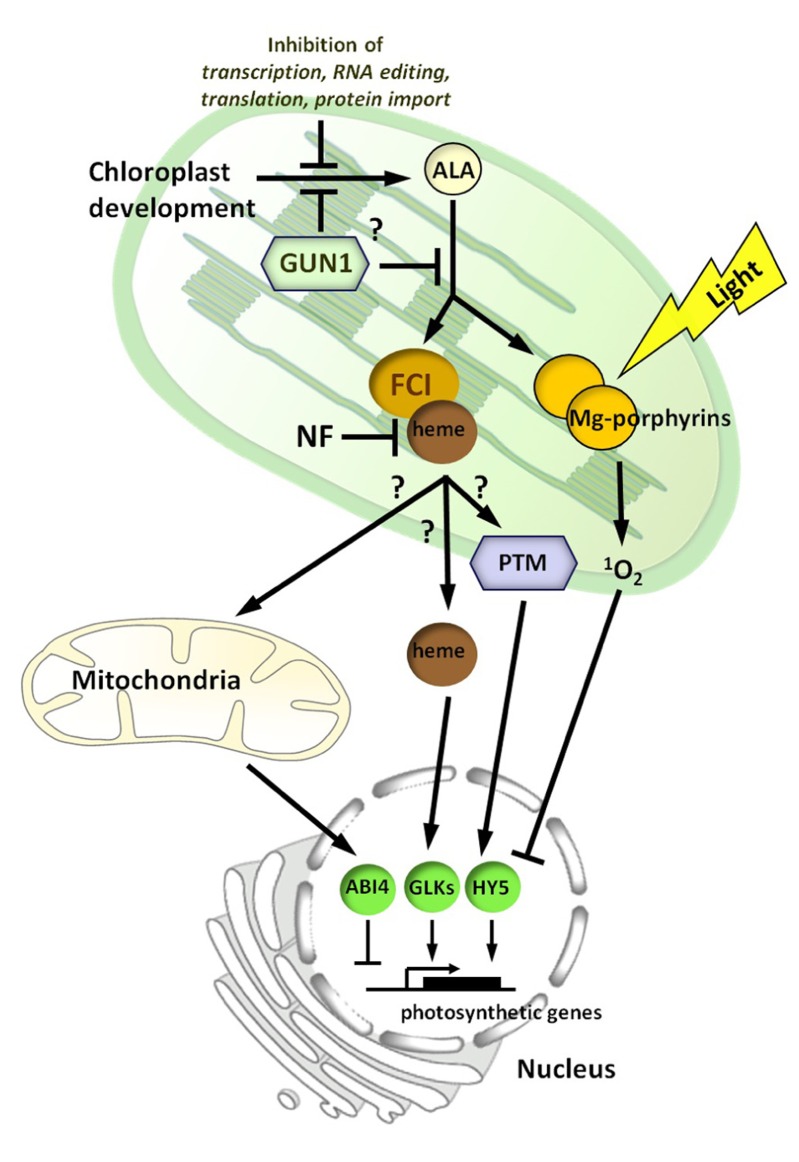
**A model for chloroplast to nucleus signaling during chloroplast biogenesis**. In this model, there are two signaling pathways proposed. A *positive* *heme-related *signal, mediated by ferrochelatase 1 (FC1), whose production reflects the developmental status of the chloroplast. Inhibition of chloroplast development by norflurazon (NF) treatment blocks production or transmission of this signal. The heme-related signal may be heme itself or a heme metabolite, and is possibly conveyed via the mitochondria. The plant homeodomain transcription factor (PTM) is also a good signaling candidate and a number of other nuclear-localized transcription factors, including ABI4, HY5, and GLK1/2 have been implicated. GUN1 is proposed to repress chloroplast development or the synthesis of the signal before or after the synthesis of the rate-limiting intermediate 5-aminolevulinic acid (ALA). The environment can impact adversely on the positive signal through effects on chloroplast development. Under conditions in which this leads to excess accumulation of chlorophyll precursors, such as Mg-porphyrins, a second *inhibitory light-dependent *signal, mediated by singlet oxygen (^1^O_2_), represses photosynthetic and tetrapyrrole synthesis genes.

A transcriptomic analysis of the effect of a FR pre-treatment on nuclear gene expression showed a strong overlap with a singlet oxygen-regulated gene set (McCormac and Terry, unpublished results). We therefore propose that the positive, inductive heme-related pathway is balanced by a repressive/inhibitory signaling pathway that is initiated by singlet oxygen generated via the accumulation of Mg-porphyrins and other chlorophyll precursors (**Figure 3**). This repressive signal may not have been observed in transcriptomic experiments using *flu, *which also accumulates Mg-porphyrins*, *as these studies used *flu* plants at the rosette stage (op den Camp et al., 2003). In contrast to the positive signal, which can be demonstrated to function in the dark (Sullivan and Gray, 1999; Woodson et al., 2013), the repressive signal is light-dependent, consistent with its role in protection from photo-oxidative damage. The primary aim of this repressive pathway is to shut-down tetrapyrrole synthesis (and chloroplast development) to prevent seedling lethality under conditions in which regulation of the tetrapyrrole pathway has been compromised. In this respect, the FR pre-treatment may be representative of seedling emergence under leaf litter, or by an extended dark period. Certainly mutants in light signaling pathways such as the *pif* mutants also show similar responses (Huq et al., 2004; Stephenson et al., 2009), and thus any perturbation of the light or developmental pathways regulating etioplast and early chloroplast development may need to be compensated for by a protective repressive signaling pathway. In this regard, it is interesting that the plant goes to great lengths to balance the synthesis of protochlorophyllide and POR through the combined action of light and hormone signaling pathways to optimize this de-etiolation response (Zhong et al., 2009; Cheminant et al., 2011).

## THE ROLE OF GUN1

The development of a model in which two different tetrapyrrole-related pathways regulate the expression of photosynthesis-related genes might explain some of the observations in the literature that are difficult to reconcile with existing models. This includes the role of GUN1. Because the *gun1* mutant rescues plastid signaling under a wide range of conditions that reduce nuclear gene expression, including treatment with NF and Lin (Koussevitzky et al., 2007), or the *sig2* (Woodson et al., 2013), and *ppi2* (Kakizaki et al., 2009) mutations, models of GUN1 function have invariably placed it in a central integrating role *downstream* of these supposed signals. However, we suggest an alternative model that fits the existing data in which GUN1 functions *upstream* of the heme-related signal, as either a general repressor of chloroplast development or more specifically as a repressor of tetrapyrrole accumulation. Thus in a *gun1* mutant the positive heme-related signal would be enhanced. Indeed such an effect was observed on the reduced heme levels seen in *sig2* (Woodson et al., 2013). The extension of this argument is that *gun1* mutants would be expected to have an increased positive signal in the dark (as the repressive signal is light-dependent). This is observed in the *sig2* mutant (Woodson et al., 2013) and we have also reported that the expression of the plastid signal-responsive gene *HEMA1* is increased in the dark in *gun1* seedlings (McCormac and Terry, 2004). Finally, an increase in tetrapyrrole accumulation should also increase the strength of the inhibitory signal under appropriate conditions and *gun1* seedlings are more susceptible to loss of gene expression following a FR treatment (McCormac and Terry, 2004). The role of GUN1 as a repressor of chloroplast development would appear at first to be in contradiction to earlier studies suggesting it is required for chloroplast development (Mochizuki et al., 1996; Ruckle et al., 2007). This interpretation rests on the observation that *gun1* seedlings do not green normally in the light. However, we would argue that this inability to green is completely consistent with an elevated *HEMA1* expression and increased tetrapyrrole synthesis which would lead to an increased susceptibility to photobleaching on transfer to white light (Mochizuki et al., 1996; McCormac and Terry, 2004). In this respect the situation is very similar to the interpretation of the *pif3* mutant phenotype. In this case the inability to green on transfer to light was originally interpreted as PIF3 having a positive role in chloroplast development (Monte et al., 2004), but it was subsequently demonstrated that PIF3 functions to repress photosynthetic gene expression (Stephenson et al., 2009).

What might be the function of GUN1 in repressing chloroplast development or tetrapyrrole synthesis? GUN1 encodes a PPR protein (Koussevitzky et al., 2007). PPR proteins bind RNA and are involved in RNA metabolism, resulting in changes in plastid gene expression (Schmitz-Linneweber and Small, 2008). One obvious target for GUN1 might therefore be the plastid-transcribed tRNA^Glu^, the substrate for glutamyl-tRNA reductase (**Figure 2**), the rate limiting step of tetrapyrrole synthesis. If the role of GUN1 was to control the availability of tRNA^Glu^ for heme synthesis then its absence might lead to increased plastid signaling via increased substrate for FC1, and therefore a stronger heme signal under conditions when this is normally limited. The recent demonstration that the sigma factors SIG2 and SIG6 are both required for nuclear gene expression, tRNA^Glu^ expression and heme synthesis, support this link (Woodson et al., 2013). However, although *gun1* could rescue the gene expression phenotype of *sig2* and *sig6* it did not do this through an increase in steady-state levels of tRNA^Glu^ transcript (Woodson et al., 2013). Instead it is possible to speculate about other mechanisms of GUN1 function such as restricting access of tRNA^Glu^ to glutamyl-tRNA reductase, rather than protein synthesis, or even selection between the glutamyl-tRNA reductase isoforms encoded by the *HEMA1* and *HEMA2* genes. Alternatively GUN1 may have a broader role in plastid RNA metabolism. Kakizaki et al. (2012) observed that many conditions that affect plastid signaling also affect RNA editing in plastids, although no differences in editing were seen in *gun1* for the two genes studied, *accD* and *rps14*. It has been noted that GUN1 shares considerable similarity with pTAC2 (Koussevitzky et al., 2007), which forms part of a transcriptionally active complex required for expression of PEP-transcribed genes (Pfalz et al., 2006). Interestingly, another component of this complex, pTAC12 (HEMERA), has dual chloroplast and nuclear localization (Chen et al., 2010), and plays an important role in light signaling (Galvão et al., 2012). Given the strong interaction between plastid and light signaling (Ruckle et al., 2007; Larkin and Ruckle, 2008), understanding the link between PEP-mediated plastid transcription and light regulation of nuclear gene expression may also provide further clues about the role of GUN1.

In contrast to *gun1*, the *gun2–gun6* mutants cannot rescue conditions that block early chloroplast development. An explanation for this is that these mutations “protect” the loss of the heme signal by increasing heme synthesis at the expense of Mg-porphyrin or bilin production, but in treatments affecting the early stages of chloroplast development there is no signal to protect. Similarly, the model explains the rather unusual observation that, while Lin and NF treatments or FR treatment and NF are additive, Lin partially rescues a FR treatment response (McCormac and Terry, 2004). Our model would propose that because Lin treatment blocks chloroplast development it prevents Mg-porphyrin synthesis and the full expression of the inhibitory response. Moreover, it is possible that, since a singlet oxygen signal would be produced by excitation of tetrapyrroles including Mg-proto, the inhibitory pathway may account for some of the studies in which a correlation between Mg-proto accumulation and inhibition of nuclear gene expression has been observed (Strand et al., 2003; Pontier et al., 2007; Kindgren et al., 2011): the fact that both pathways are operating under some conditions and that individual genes show different sensitivities to treatments affecting them (McCormac and Terry, 2004) may also explain why this correlation is far from absolute (Mochizuki et al., 2008; Moulin et al., 2008; Voigt et al., 2010). The same argument could also account for some of the confusion about whether GUN1 and GUN2–GUN6 function in the same pathway (e.g., Vinti et al., 2000; Mochizuki et al., 2001; McCormac and Terry, 2004).

## HOW DO TETRAPYRROLES SIGNAL TO THE NUCLEUS?

The most significant gap in our understanding of plastid signaling is the nature of the signal that moves from the plastid to influence nuclear gene expression, either by acting in the nucleus directly or via interaction with a cytoplasmic signaling pathway. It is possible that heme derived from FC1 can function as a mobile signal to interact directly with downstream components in the nucleus or cytoplasm. Heme is less photo-toxic than Mg-proto and thus is more suitable as a signaling molecule, but it would nevertheless need to be associated with one of the numerous heme-binding proteins recently identified, in order to be transported around the cell (Mochizuki et al., 2010). As shown in **Figure 3**, and discussed earlier, the mitochondria could also be a destination for at least some of this pool. However, there is no requirement for heme to leave the chloroplast: any signal could be passed on immediately. An obvious candidate would be a product of heme degradation such as a bilin (Terry et al., 2002), although in this case it might be expected that *gun2* (*hy1*) and *gun3* (*hy2*; **Figure 2**) would not have *gun* phenotypes. However, the fate of heme in plant cells is still poorly understood (Mochizuki et al., 2010) and a mobile heme signal cannot be ruled out. Alternatively, with many chloroplast and mitochondrial proteins requiring a heme cofactor to be functional there are many potential mechanisms by which a heme signal could be transduced. One interesting candidate that was identified recently is the chloroplast envelope-bound PTM protein (Sun et al., 2011). Mutants in *ptm* are also *gun* mutants, and it was demonstrated that PTM functions in the same pathway as GUN1 (Sun et al., 2011). As PTM is proposed to undergo proteolytic cleavage to give an amino terminal fragment that directly regulates *ABI4* expression, such a plastid-signaling pathway would be satisfyingly simple. ABI4 has already been identified as a downstream regulator of the GUN pathway (Koussevitzky et al., 2007), where it may function to integrate a number of additional signals regulating nucleus-encoded photosynthetic genes such as sucrose (Oswald et al., 2001), ABA (Koussevitzky et al., 2007), a mitochondrial signal (Giraud et al., 2009), and most recently a heat responsive retrograde signal (Yu et al., 2012). In addition, it has been proposed that ABI4 functions together with HY5 to mediate plastid signaling responses, thus integrating them into the light signaling network (Ruckle et al., 2007; Larkin and Ruckle, 2008). However, it is likely that other transcription factors are also involved in the positive or inhibitory pathways. Two important regulators of chloroplast development, GLK1 and GLK2, are certainly candidates for such a role (Kakizaki et al., 2009; Waters et al., 2009), as are the recently described *END* genes (Ruckle et al., 2012). Whether these targets serve as a convergence point between the positive and inhibitory pathways remains to be elucidated.

If the inhibitory signal is indeed singlet oxygen-mediated, as postulated above, it is possible that its signal transduction utilizes some of the same components identified for singlet oxygen-mediated stress responses in the *flu* mutant. These include the chloroplast proteins EXECUTER1 and EXECUTER2 (EX1 and EX2; Wagner et al., 2004; Lee et al., 2007), as well as a requirement for CRY1 in a singlet oxygen-mediated programmed cell death response (Danon et al., 2006). Interestingly, CRY1 also has been proposed to play a role in response to high irradiance stress (Kleine et al., 2007), and *cry1* mutants were identified as *gun* mutants (Ruckle et al., 2007). Singlet oxygen itself has a short half-life and any information contained in a singlet oxygen burst would need to be passed locally in the first instance. It has therefore been speculated that various lipid-related metabolites of singlet oxygen action may be involved in signaling. One particularly promising candidate is the β-carotene-derived oxidation product β-cyclocitral (Ramel et al., 2012). However, it should be emphasized that an inhibitory signal involved in regulation of chloroplast development may be quite distinct from that involved in the induction of stress signaling genes. For example, EX1- and EX2-dependent cell death responses require fully developed chloroplasts to be observed (Kim et al., 2012). In addition, given the observation that singlet oxygen and hydrogen peroxide signaling appear to be antagonistic (Laloi et al., 2007) it is tempting to speculate that the induction of nuclear gene expression by the positive heme-related signal is mediated by a low level of hydrogen peroxide. In this way biogenic control might mirror the effects later in development (operational control) in which a balance of singlet oxygen and hydrogen peroxide signaling, resulting from excitation of PSII and PSI, respectively, may be important in communicating changes in relative excitation to the nucleus in mature plants. There is already considerable evidence that hydrogen peroxide functions as a plastid signaling molecule in high light responses, and so might also make a suitable candidate for retrograde signaling during chloroplast development (Galvez-Valdivieso and Mullineaux, 2010).

## CONCLUSION

In this article we have described a model for the role of chloroplast-localized tetrapyrrole synthesis in regulating nuclear gene expression. The model builds on the seminal work of the Chory laboratory in demonstrating genetically that a heme-related signal acts as a positive signal for chloroplast biogenesis (Woodson et al., 2011), but has a number of significant additions. The key features of the model are that: (i) all of the chloroplast treatments and mutants that are characterized as reducing nuclear gene expression do so by inhibiting the capacity of the chloroplast to make or process a heme-related signal; (ii) GUN1 acts early in this pathway to promote signal production, rather than late in pathway integrating many different signals; and (iii) an inhibitory signal derived from light excitation of Mg-porphyrins and other chlorophyll precursors serves to reduce tetrapyrrole synthesis and restrict chloroplast development under conditions in which these molecules are in dangerous excess. The model focuses on a single biological system, namely early seedling development in *Arabidopsis*, and thus should be easily testable and improved upon in many laboratories – once the central features of a model are defined and broadly accepted in one system, its significance across different systems can be assessed. The model focuses on what happens during biogenic control; however, a key question will be to determine the overlap between biogenic and operational control. Clearly the environment will have a major impact on early seedling development, but do developing chloroplasts sense these changes in a fundamentally different way?

There is increasing evidence that plastid signaling pathways are part of fundamental signaling networks that not only mediate the plant’s response to stress, but also operate in a pre-stress environment to co-ordinate developmental and environmental cues. For example, mutations in EX1 and EX2 inhibit singlet oxygen signaling during embryogenesis, resulting in impaired chloroplast development in germinating seedlings. In this system, the stress hormone ABA promotes chloroplast development (Kim et al., 2009) perhaps because application of ABA results in a *gun* phenotype (Voigt et al., 2010). The developing theme is that mild stress responses mediated by chloroplasts function to protect plants from more severe stress later (Saini et al., 2011). Plastid signals are linked to both environmental stresses such as high light (Galvez-Valdivieso et al., 2009; Ramel et al., 2012), cold (Yoshida et al., 1998; Yang et al., 2005b), and drought (Miller et al., 2010; Estavillo et al., 2011) as well as a range of developmental responses (Yu et al., 2007; Ruckle and Larkin, 2009; Burch-Smith et al., 2011; Fleischmann et al., 2011). How these responses are integrated with the biogenic signals described here will be a critical next step in understanding how a seedling is able to become established in a potentially hostile environment.

## Conflict of Interest Statement

The authors declare that the research was conducted in the absence of any commercial or financial relationships that could be construed as a potential conflict of interest.
